# P2X3 Receptor Ligands: Structural Features and Potential Therapeutic Applications

**DOI:** 10.3389/fphar.2021.653561

**Published:** 2021-04-13

**Authors:** Andrea Spinaci, Michela Buccioni, Diego Dal Ben, Gabriella Marucci, Rosaria Volpini, Catia Lambertucci

**Affiliations:** Medicinal Chemistry Unit, School of Pharmacy, University of Camerino, Camerino, Italy

**Keywords:** P2X3 receptor, P2X3-P2X2/3 receptor ligands, P2X3 receptor agonists, P2X3 receptor antagonists, drug discovery, Chronic cough, pain

## Introduction

**FIGURE 1:**
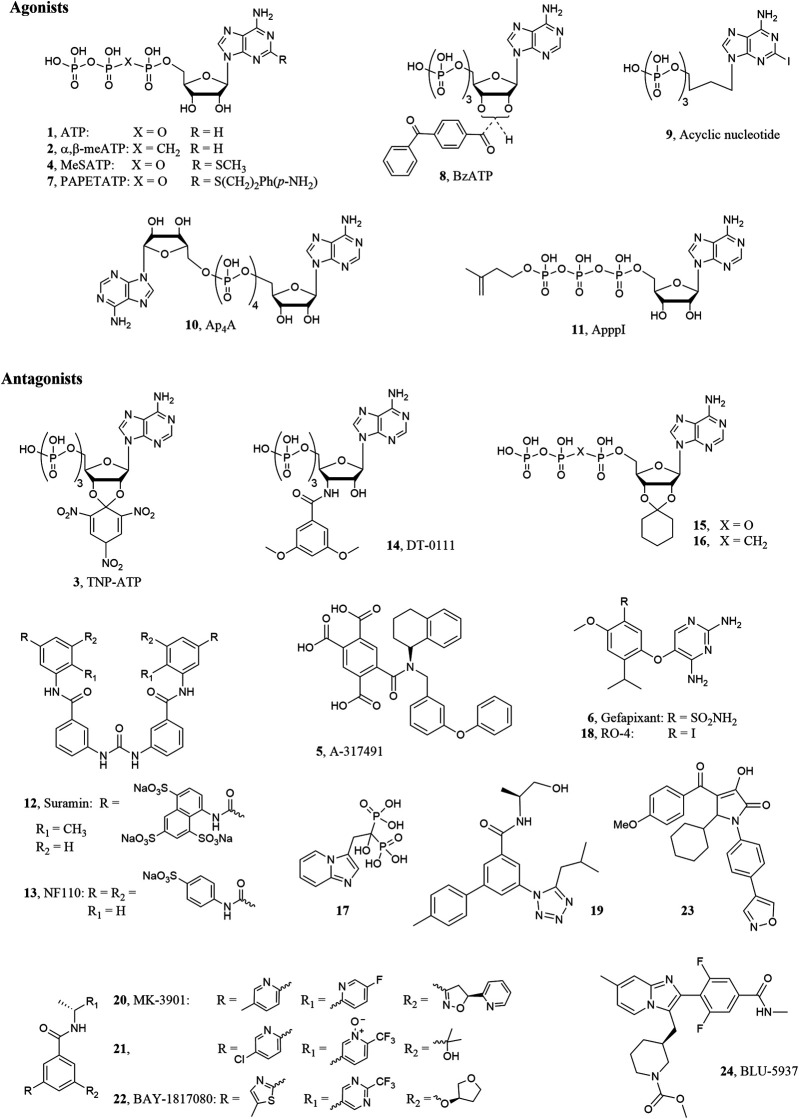
P2X3R ligand structures.

The ionotropic P2X3 receptor (P2X3R) subtype is one of the seven mammalian P2X_1-7_ receptor belonging to the P2 purinergic receptor family together with the metabotropic P2Y_1-2, 4-6,11-14_ ones (Fredholm et al., 2011). As the other P2X ion channels, it is a trimeric cell surface receptor permeable to Na^+^, K^+^, and Ca^2+^ cations and it is activated by the natural ligand adenosine-5′-triphosphate (ATP, **1**, Figure 1). Each subunit is constituted by two *trans*-membrane domains connected by a large glycosylated extracellular loop, which contains many disulfide bonds and the ATP binding site (Browne et al., 2010). P2X3Rs are assembled as homotrimers, constituted by three subunits of P2X3Rs, or heterotrimers, constituted by two P2X3Rs and one P2X2R subunits (P2X2/3Rs) (Lewis et al., 1995). A difference between the two forms is represented by their fast or slow desensitization after prolonged exposure to agonists; hence P2X3Rs undergo rapid inactivation/desensitization during exposure to ATP or to the selective agonist α,β-methyleneATP (α,β-meATP, **2**, Figure 1), which is accelerated by increasing the agonist dose, while P2X2/3Rs shows either mixed (two-component) or slow-type desensitization (Giniatullin and Nistri, 2013).

## P2X3 Receptors as Target for Potential Drugs

Both the homotrimeric P2X3 and heteromeric P2X2/3Rs are expressed on terminals of primary afferent sensory neurons of dorsal root ganglia (DRG), spinal cord and brain and their activation by ATP mediates pain sensation, hyperalgesia and allodynia in rodents (Jarvis, 2003). Notably, unlike rodents, the P2X3 subtype is the predominant ATP receptor subtype in human DRG sensory neurons (Serrano et al., 2012). On the other hand, the expression of the P2X2/3 heteromers is higher in afferents of viscera with respect to the somatic innervation, leading to potential different therapeutic approaches to treat visceral or somatic pain (with a P2X3 selective or a dual P2X3-P2X2/3 antagonist) (Ford, 2012). The injection of ATP or α,β-meATP into rodent skin induced nociceptive behavior (Kennedy, 2005; De Logu and Geppetti, 2019), whereas the use of the P2X3R antagonist 2′,3′-O-(2,4,6-trinitrophenyl)adenosine-5′-triphosphate (TNP-ATP, **3**, Figure 1) mediated antinociceptive effects (Honore et al., 2002). Hence, these receptors represent attractive targets to treat pain related disorders (Burnstock, 2018). P2X3 and P2X2/3Rs are functionally expressed also into the lamina propria, urothelium and detrusor smooth muscle of urinary bladder (Ford and Cockayne, 2011). It has been demonstrated that ATP or α,β-meATP dose dependently stimulated bladder overactivity in conscious rats and this effect was antagonized by TNP-ATP. Hence, P2X3 and P2X2/3R antagonists are recognized as potential drugs to treat urological dysfunction, such as overactive bladder (Ford et al., 2006; Andersson, 2016). It is worthwhile to note that extracellular ATP, released in terminals of primary afferents in airway tissue or at the level of their central synapses, mediates protective reflex responses including cough, through interaction with P2X3 and P2X2/3Rs (Weigand et al., 2012). This finding constitute the rationale for the use of these receptors antagonists in patient affected by chronic obstructive pulmonary disease (COPD) and chronic cough (Dicpinigaitis et al., 2020).

## P2X3 Receptor Ligands

Most of the P2X3 agonists reported so far derived from modification of the ATP molecule, however, the search for molecules that activate these receptors is mainly of experimental interest. Due to the above mentioned therapeutic applications, in the last years many efforts have been directed through the discovery of potent and selective P2X3 and P2X2/3R antagonists derived both from high throughput screening of large compound libraries and rational design by molecular modeling studies, which were facilitated by the publication of the crystallographic structures of the P2X receptors. Beside *in silico* studies carried out with the development of theoretical 3D models of the P2X3 receptor (Grimes and Young, 2015). more recent works were based on experimental structures of the same protein. At present, several X-Ray structures of the P2X3 receptors are available in complex with agonists, i.e. ATP or 2-methylthioATP (2-MeSATP, **4**), and ATP competitive (orthosteric) antagonists like TNP-ATP and 5-[[[(3-phenoxyphenyl)methyl][(1*S*)-1,2,3,4-tetrahydro-1-naphthalenyl]amino] carbonyl]-1,2,4-benzenetricarboxylic acid (A-317491, **5**) or non-competitive (allosteric) antagonists like 5-(2,4-diamminopyrimidin-5-yloxy)-4-isopropyl-2-methoxybenzenesulfonamide (AF-219, also called MK-6274 or gefapixant, **6**, Figure 1) (Mansoor et al., 2016; Wang et al., 2018; Li et al., 2019).

### Agonists

The natural ligand ATP (**1**; EC_50_ = 0.5 μM) (Jarvis and Khakh, 2009) is able to activate P2XRs with various degrees of affinity. Extracellular ATP is rapidly hydrolized by ecto-nucleotidas to form ADP, AMP, and then adenosine. Compared to ATP, ADP show weaker activity at P2X3Rs (Lewis et al., 1995; North and Surprenant, 2000). Chemical modification of ATP led to P2X3R agonists and also antagonists (Lambertucci et al., 2015). In particular, the introduction of substituents in the 2-position of ATP slightly increased the P2X3R affinity leading to the unselective agonist 2-MeSATP (**4**; EC_50_ = 0.35 μM) (Jarvis and Khakh, 2009), which binds also the other P2X subtypes, especially the P2X1Rs. On the contrary, the 2-substituted *p*-aminophenylethylthioATP (PAPET-ATP, **7**; EC_50_ = 0.017 μM) resulted a potent and selective P2X3R agonist at rat receptor (Jacobson et al., 2006). Recent experiments of X-Ray crystallography were reported showing the binding mode of both ATP and 2-MeSATP at the human P2X3R, with information about the roles of the triphosphate chain and the 2′- and 3′-hydroxyl groups in the ligand-target interaction (Mansoor et al., 2016; Li et al., 2019). Since the triphosphate chain of the ATP derivatives is easily and rapidly hydrolyzed by ectonucleotidases, stable analogues like α,β-meATP, which activate P2X3Rs with EC_50_ in the sub μM range, were synthesized. Modification of the ATP sugar moiety resulted in the potent agonist benzoylATP (BzATP, **8**; EC_50_ = 0.08 μM) (Jarvis and Khakh, 2009), which is a mixture of the 2′- and 3′-benzoyl esters, while its replacement with open chains gave acyclic nucleosides like 2-iodo-9-butyladenine triphosphate (**9**; EC_50_ = 0.08 μM), which showed responses of 60% of the maximal effect elicited by α,β-meATP in patch-clump assay, so behaving as partial agonist (Volpini et al., 2009).

Some naturally occurring diadenosine polyphosphates were found as unselective P2X agonists with micro- or submicromolar activities, with the tetra- and triphosphonate derivatives Ap_4_A (**10**, Figure 1) and Ap_3_A showing full and partial rP2X3R agonist profile, respectively (both molecules being endowed with higher potency than ATP) (Wildman et al., 1999). Ap_4_A showed ability to readily desensitize hP2X3R at nanomolar concentrations (McDonald et al., 2002). In a recent work, chemically stable analogues of this molecule showed a similar ability to desensitize the human and rat P2X3Rs, with a weak partial agonist profile only at high concentrations (Viatchenko-Karpinski et al., 2016). Chemical modifications of the diadenosine polyphosphates led to development of the so-called nitrogen-containing bisphosphonates (NBPs), which are of clinical relevance in particular for bone diseases. Among these molecules, ApppI (**11**, Figure 1) showed to rapidly desensitize rat P2X3R at low nanomolar concentration (Ishchenko et al., 2017). The high-potency activation of the P2X3 by these molecules, followed by rapid desensitization, makes them *de facto* inhibitors of this receptor, with a clinical potential as analgesics.

### Antagonists

While most of the P2X3 agonists reported so far derived from modification of the ATP molecule, the antagonists belong to various chemical classes. The first orthosteric antagonists to be identified were the polysulphonated naphthylurea suramin, its derivatives and various histochemical dyes (Jacobson et al., 2002). Suramin (**12**; IC_50_ = 3.0 μM) (Lewis et al., 1995) and its derivatives behave as nonselective antagonists with IC_50_ in the micromolar range at P2X3Rs, with the exception of NF110 (**13**, IC_50_ = 0.09 μM), which displayed submicromolar activity at rat receptors. The ATP derivative 3′-deoxy-3’-(3,5-dimethoxybenzamido)ATP (DT-0111, **14**; IC_50_ 3.0 μM), bearing a dimethoxyphenyl amido group in place of the hydroxyl in 3′-position of the ribose, is recognized as a novel small water soluble molecule that behaves as a selective antagonist at P2X2/3Rs. When administered as an aereosol in *in vivo* experiments, the compound inhibited bronchoconstriction and cough induced by ATP (Pelleg et al., 2019; Illes et al., 2020). The linking of the 2′ and 3′ ATP hydroxyl groups by a trinitrophenyl ring gave the compound TNP-ATP (**3**; IC_50_ = 0.001 μM), which resulted the most potent competitive antagonist with potency in the low nanomolar range (Lewis et al., 1998) (Lambertucci et al., 2015). Molecular modeling studies led to the design of derivatives in which the trinitrophenyl group of TNP-ATP was replaced by cycloalkyl or aromatic rings (Dal Ben et al., 2015; Dal Ben et al., 2017). Among them, the 2′,3′-*O*-cyclohexylideneATP (**15**, Figure 1) displayed an IC_50_ = 0.083 μM. Modification of the triphosphate chain of this derivatives gave the α,β-methylene stable analogue **16**, which displayed an IC_50_ = 17.5 μM at human and 0.127 μM at rat P2X3Rs, being selective vs. the other P2X subtypes (Dal Ben et al., 2019). The substitution of the cycloalkyl group of **15** with a small alkyl moiety maintained the antagonist activity, with the isopropylidene function being the smallest group allowed (Dal Ben et al., 2019).

The small non-nucleotide molecule A-317491 (**5**; IC_50_ = 0.02 μM) was the first identified potent and selective P2X3 and P2X2/3R competitive blocker. However, although in its structure are present three carboxylic groups, it is endowed with low water solubility and oral bioavailability (Jarvis et al., 2002). X-Ray crystallography experiments showed that A-317491 and TNP-ATP bind in the same cavity of ATP hence confirming the ATP-competitive mechanism of action, with the tetracarboxylic acid moiety of A-317491 occupying the same position adopted by the triphosphate chain of TNP-ATP (Mansoor et al., 2016).

Few years later, medicinal chemistry efforst by AstellasPharma led to the development of imidazopyridine derivatives with activity on P2X2/3 receptors (Kakimoto et al., 2008). Among these compounds, minodronate (**17**, Figure 1) was approved to market in Japan for the treatment of osteoporosis given its ability to inhibit of farnesyl pyrophosphate synthase; its additional activity on P2X2/3 resulted an advantage to reduce low back pain in patients (Ohishi and Matsuyama, 2018).

Later on, a number of diaminopyrimidine derivatives were reported by the company Roche as potent and selective negative allosteric modulators of P2X3 and P2X2/3Rs like the 5-(5-iodo-2-isopropyl-4-methoxyphenoxy)pyrimidine-2,4-diamine (RO-4 or AF-353, **18**; IC_50_ = 3.16 nM) or gefapixant (**6**; IC_50_ of 0.03 and 0.250 μM at P2X3 and P2X2/3Rs, respectively), which are endowed with favorable pharmacokinetic profile (Carter et al., 2009; Gever et al., 2010; Ford and Undem, 2013). X-Ray crystallography experiments showed that gefapixant binds the P2X3 at the interface between the receptor subunits, in a different binding pocket than ATP. Interestingly, the substituted benzyl moiety of this molecule occupies a sub cavity that is partially occupied also by the trinitrophenyl group of TNP-ATP (Mansoor et al., 2016; Wang et al., 2018). Replacement of the pyrimidine scaffold of these molecules with a purine led to derivatives that, although less potent, retained the ability to block the receptors (Lambertucci et al., 2013). Given the interesting therapeutic potential of molecules that block P2X3 and P2X2/3Rs, in recent years a number of antagonists endowed with a good pharmacokinetic profile and reasonable oral bioavailability have been discovered and reported in numerous patents by pharmaceutical companies (Marucci et al., 2019). Among the diaminopyrimidine derivatives, the most promising one reported from Roche and developed by Merck is the above cited gefapixant (**6**), which was studied in clinical trials for different pathologies like idiopathic bladder disorders, osteoarthritic joint pain, and pulmonary cystic fibrosis (Jarvis, 2021). In patients with refractory chronic cough, gefapixant dose dependently reduced awake cough frequencies, however it induced alteration of taste sensitivity (dysgeusia) and, in some cases, the completed loss of taste (ageusia). The loss of taste response seems to be due to its unselective block of P2X3 and P2X2/3Rs. Although these side effects, the medical need for patients suffering of this pathology, encouraged Merck to continue the development of gefapixant, which has recently completed two Phase III clinical trials (called COUGH-1 and COUGH-2; NCT03449134, 2020; NCT03449147, 2020) for refractory or unexplained chronic cough (Muccino et al., 2020). In these studies, gefapixant at the dose of 45 mg twice daily induced a significant reduction in 24-h cough frequency with mild to moderate alteration of taste sensitivity (Merck News release, 2020).

Also arylamide derivatives were found as potent and selective P2X3R antagonists. In particular, the 5-(5-isobutyltetrazol-1-yl)-4′-methylbiphenyl-3-carboxylic acid ((*S*)-2-hydroxy-1-methylethyl)amide (**19**; pIC_50_ = 8.8), reported by Roche company, resulted the most active compound at P2X3Rs in *in vitro* functional experiment performed using FLIPR (Fluorometric Imaging Plate Reader) Assay (Dillon et al., 2015). Another potent and selective P2X3R antagonist belonging to the arylamide derivatives was found by Merk. This compound, named MK-3901 (*N*-[1(*R*)-(5-fluoropyridin-2-yl)ethyl]-3-(5-methylpyridin-2-yl)-5-[5(*S*)-(2-pyridyl)-4,5-dihydroisoxazol-3-yl]benzamide, **20**), was tested in a Ca^2+^ mobilization FLIPR assay (FLIPR IP = 21 nM) and showed an efficacy comparable to that of naproxen in a rat inflammatory model and a very good bioavailability in different species. However, it was shown to induce hyperbilirubinemia in preclinical studies, so it was modified in order to avoid this effect and to improve its pharmacokinetic properties and *in vivo* potency. In fact, in a preclinical inflammatory pain model, MK 3901 showed and EC_90_ = 3 μM while its improved derivative **21** exerted an EC_90_ of 0.16 μM (Ginnetti et al., 2018). The allosteric potent and selective P2X3R antagonist belonging to arylamide derivatives 3-(5-methylthiazol-2-yl)-5-(((*R*)-tetrahydrofuran-3-yl)oxy)-*N*-((*R*)-1-(2-(trifluoromethyl)pyrimidin-5-yl)ethyl)benzamide (eliapixant also called BAY-1817080, **22**; IC_50_ = 8 nM), developed by Bayer, showed a dose dependent reduction in cough frequency with low effect on taste perception in a phase IIb clinical trials. Very recently for this compound the company planed Phase II clinical trials for different pathology like diabetic neuropathies and overactive bladder and Phase I clinical trial for endometriosis (NCT04545580, 2020; NCT04614246, 2020).

Also pyrrolinone derivatives have been identified as a novel class of P2X3 receptor antagonists by Shionogi company. Among them, the 5-cyclohexyl-3-hydroxy-1-(4-(isoxazol-4-yl)phenyl)-4-(4-methoxybenzoyl)-1,5-dihydro-2*H*-pyrrol-2-one (**23**) resulted a very potent antagonist of P2X3Rs with an IC_50_ of 0.025 μM and a good analgesic efficacy in an acetic acid induced writhing test. Its selectivity was proved respect to 41 receptors and 17 enzymes (Tobinaga et al., 2018).

Among selective P2X3R antagonists there are novel imidazo-pyridine developed by Biopharmaceutical Company BELLUS Health with the aim to avoid the unpleasant loss of taste which characterized P2X3 and P2X2/3Rs unselective ligand. The most promising seemed to be methyl (*S*)-3-((2-(2,6-difluoro-4-(methylcarbamoyl)phenyl)-7-methylimidazo [1,2-a]pyridin-3-yl)methyl)piperidine-1-carboxylate (BLU-5937, **24**; IC_50_ of 0.025 μM and >24 μM at human P2X3 and P2X2/3Rs, respectively) (Pharmacompass, 2018), a compound that demonstrated a good oral availability and pharmacokinetic profile. Differently from gefapixant, BLU-5937 did not affect taste function, even at high doses, in a two bottle taste study (Garceau and Chauret, 2019). The lack of this side effect was demonstrated also in a Phase II clinical study, however it didn’t reach the primary endpoint since it did not achieve statistical significance, at any dose tested, for the reduction of cough frequency vs. placebo (Evaluate Ltd, 2020). BLU-5937 is currently under clinical evaluation (phase II) for the treatment of chronic pruritus in adult subjects with atopic dermatitis (NCT04693195, 2021).

Another selective P2X3 allosteric blocker, S-600918, developed by Shionogi (structure not reported), is currently being studied in Phase II clinical trials in patients suffering from refractory chronic cough (Dicpinigaitis et al., 2020; Jacobson et al., 2020). This compound showed to induce a reduction of cough frequency vs. placebo with moderate alteration of taste sensitivity in a Phase IIa (JapicCTI-184027, 2019) clinical trial (Niimi et al., 2019). Recruitment is already terminated for the Phase IIb (NCT04110054, 2020) clinical trial, consistent in a randomised, double-blind, placebo-controlled study to assess the onset of efficacy and determine the optimal dose (Ishihara et al., 2020).

## Discussion

The limited distribution of P2X3 and P2X2/3Rs and their role in mediating painful stimuli in the primary sensory neurons of DRG make these receptors attractive targets in different pathologies. In fact, P2X3 and P2X2/3R antagonists, which inhibit ATP mediating effects on these receptors, represent very interesting tools that could lead to potential analgesic drugs. The advantage of these molecules is their peripheral action, which avoids sedation, gastrointestinal or cardiovascular side effects typical of the current analgesic drugs. In fact, while the availability of selective agonists of these receptors is still a challenge, in recent years many research efforts have led to the discovery of orthosteric and allosteric antagonists belonging to different chemical class. The availability of reliable experimental 3D structures of the P2X3R is a critical factor for the depiction of the mechanism of action of known orthosteric and allosteric ligands and for computational studies aimed at virtually screening or structure-based designing novel potential modulators. Nevertheless, for some compounds endowed with an allosteric mechanism of inhibition and structurally unrelated to the co-crystallized compound gefapixant, the structural determinants at the basis of their interaction with the receptor are still unknown. Molecular modeling studies or further X-Ray crystallography or electron cryomicroscopy experiments could be of help to depict their mechanisms of action and for the design of novel inhibitors.

Compared to marketed orally bioavailable analgesic drugs, the first generation of P2X3 antagonists preclinically showed a not suitable drug-like profile based on their poor P2X subtype selectivity and/or unfavorable pharmacokinetic/pharmacodynamic profile (i.e. limited adherence to the Lipinski rules, toxicity, interaction with other drugs) (Gum et al., 2012). These factors compromised their clinical development. An example is given by the first P2X3 antagonist preclinical candidate discovered by Merck, MK-3901 (**20**, Figure 1). As already mentioned, in preclinical studies this molecule showed relevant side effects (hyperbilirubinemia), low metabolic stability, and cytochrome P450 inhibition potentially leading to drug-drug interaction (Ginnetti et al., 2018).

More recent P2X3 antagonists were hence developed with improved chemical-physical properties to achieve a drug-like profile combined with high efficacy at the P2X3 receptors. At present, four P2X3 and P2X2/3R allosteric antagonists are under evaluation in different phases of clinical trials for the treatment of patients suffering of overactive bladder, diabetic neuropathies, endometriosis, and refractory chronic cough. For this last pathology, it seems that unselective ligands which bock both the P2X3 and P2X2/3Rs led to a loss of taste as undesirable side effect that seems to be avoided with the use of selective P2X3R antagonists. In fact, the selective allosteric blocker BLU-5937 did not affect taste function, however failed to reach its primary point. Hence, the selective blocker eliapixant or the unselective antagonist gefapixant, which has recently completed two Phase III clinical trials could be the new approved drug for the treatment of refractory chronic cough.

## Perspectives

Treatment of pain associated to diseases is still a challenge, and a small percentage of sufferers receive adequate prescription for treatment. The restricted localizations of P2X3 and P2X2/3Rs on nociceptive neurons make them an attractive target for the management of inflammatory, neuropathic, and visceral pain states and chronic and refractory chronic cough. The lack of central, gastrointestinal and cardiovascular effects of molecules that block these receptors has greatly stimulated the synthesis of P2X3R antagonists. Most of these molecules are allosteric modulators and some of them are under evaluation in clinical trials. The most relevant side effect of gefapixant is the dysgeusia, which is attenuated or absent in the case of eliapixant and BLU-5937. For the refractory and chronic cough BLU-5937 did not reach its primary end point while gefapixant, although its effect on taste perception, completed phase III clinical trial. Blu is at present evaluated in clinical trial for endometriosis associated pain and chronic pruritus associated atopic dermatitis while eliapixant is under evaluation for endometriosis and chronic cough. We are confident that in the next future these molecules will reach the market as new analgesic and antitussive drugs.
